# Antioxidant, cytotoxic and apoptotic activities of the rhizome of *Zingiber zerumbet* Linn. in Ehrlich ascites carcinoma bearing Swiss albino mice

**DOI:** 10.1038/s41598-022-15498-8

**Published:** 2022-07-15

**Authors:** Hanif Ali, Rumana Yesmin Hasi, Majidul Islam, Md Shajedul Haque, Mustfa F. Alkhanani, Atiah H. Almalki, Shafiul Haque, R. Z. Sayyed, Tanzima Yeasmin

**Affiliations:** 1 Department of Biochemistry and Molecular Biology, University of Rajshahi, Rajshahi, 6205 Bangladesh; 2 Biology Department, College of Sciences, University of Hafr Al Batin, P. O. Box 1803, Hafr Al Batin, 31991 Saudi Arabia; 3 Department of Pharmaceutical Chemistry, College of Pharmacy, Taif University, P.O. Box 11099, Taif, 21944 Saudi Arabia; 4 Addiction and Neuroscience Research Unit, College of Pharmacy, Taif University, Al-Hawiah, Taif, 21944 Saudi Arabia; 5 Research and Scientific Studies Unit, College of Nursing and Allied Health Sciences, Jazan University, Jazan, 45142 Saudi Arabia; 6 Faculty of Medicine, Bursa Uludağ University, Görükle Campus, 16059 Nilüfer, Bursa, Turkey; 7 Department of Microbiology, PSGVP Mandal’s S I Patil Arts, G B Patel Science and STKVS Commerce College, Shahada, 425409 India

**Keywords:** Antimicrobials, Biotechnology

## Abstract

Due to having a long history of traditional uses as a functional food, *Zingiber zerumbet* was selected here to explore the inherent antioxidant and antineoplastic activities of methanolic extract of its rhizome (MEZZR) against Ehrlich ascites carcinoma (EAC) cells. The rich polyphenol containing MEZZR showed a marked DPPH, ABTS, nitric oxide radicals and lipid peroxidation inhibition activity with an IC_50_ of 3.43 ± 1.25, 11.38 ± 1.39, 23.12 ± 3.39 and 16.47 ± 1.47 µg/ml, respectively, when compared to the standard catechin. In vivo, MEZZR significantly inhibited EAC cell growth, decreased body weight gain, increased life span and restored the altered hematological characteristics of EAC-bearing mice. Moreover, MEZZR induced nuclear condensation and fragmentation, which are notable features of apoptosis as observed by fluorescence microscopy after staining EAC cells of MEZZR-treated mice with Hoechst 33342. Additionally, in vitro, the cell growth inhibition caused by the MEZZR in MTT assay, was remarkably decreased in the presence of caspase-3, -8 and -9 inhibitors. This study thus suggests that MEZZR may possess promising antiproliferative efficacy against EAC cells by inducing cell apoptosis.

## Introduction

Free radicals are predominantly the leading cause of several disorders in the human body that are continuously generated as an imbalance between the formation and neutralization of reactive species. Oxidative stress is initiated by free radicals and causes excessive damage to tissues, and lipids, proteins, and DNA, leading to various chronic diseases like cancer, atherosclerosis, diabetes, cardiovascular disease, ageing and inflammatory diseases^1,2^. Endogenous enzymes (superoxide dismutase, catalase) and antioxidants (ascorbic acid, tocopherol, and glutathione) are the most common ways in which the human body defends itself from oxidative stress^3^. However, these endogenous molecules are not always safeguarded against oxidative-induced damage to the body. On the other hand, exogenous antioxidants are applicable to combat this oxidative damage^4–6^. Therefore, researchers have given importance to finding out which ethnomedicines have potent antioxidant activities with diminished toxicity.

As one of the principal causes of health-related disorders, cancer has dominated in both developing and developed countries. The number of new cases is supposed to increase by about 70% over the next twenty years^7^. Chemotherapeutic agents are still considered as one of the treatment strategies for cancer. Still, their use is limited due to high financial costs, suppression of bone marrow, gastrointestinal lesions, drug resistance, neurologic dysfunction, and cardiac toxicity in multiple organ systems^8^. Therefore, necessary actions must be taken to develop new anticancer drugs. Recently, medicinal plants and their products are always considered abundant in bioactive phytochemicals with minimal side effects. These phytochemicals have several pharmacological properties, including antineoplastic and antioxidant effects^9–11^. Moreover, natural plant-based bioactive compounds exhibit their antiproliferative action against cancer cells, but not on host cells^12^. Therefore, investigating medicinal plants to explore bioactive compounds with strong anticancer activities with fewer or no side effects is urgently needed.

As an experimental tumor, Ehrlich ascites carcinoma (EAC) cells are rapidly growing within the peritoneal cavity of mice with very aggressive behavior. EAC cells are convenient for anticancer drug tests due to their suitability for study in a mice mode and their similarity to many human tumors. Moreover, the lack of H-2 histocompatibility antigen within EAC cells is one of the probable reasons for their quick proliferation^13^. For that reason, EAC cells are very suitable for evaluating the anticancer effects of plant extracts in mouse models.

*Zingiber zerumbet*, commonly known as Bon ada, belongs to the Zingiberaceae family and is widely grown in both tropical and subtropical areas of the world^14^. The rhizome of this plant was found to be the most crucial part which has been traditionally used to prevent various diseased conditions such as inflammation, diarrhea, stomach cramps, bacterial infections, fever, flatulence, allergies, and poisoning^15–18^. Ethanol extract of this rhizome has been found to possess a good amount of polyphenolic compounds and various pharmacological properties including antioxidant, antimalarial, antibacterial and antifungal properties^19–21^. *Zingiber zerumbet* contains a predominant sesquiterpene, zerumbone, which has significant protective activity against several human cancers^22,23^. However, the literature review revealed no reports on the combined antioxidant and antitumor activities of the rhizome of the *Zingiber zerumbet* plant.

Herein, the methanolic extract of the rhizome of *Zingiber zerumbet* (MEZZR) was evaluated for antioxidants, cytotoxic and antitumor activities using various in vitro and in vivo studies. In addition, the study also focused on the chemical composition of MEZZR.

## Results

### Chemical profile of MEZZR analyzed by GC–MS

**Figure 1:**
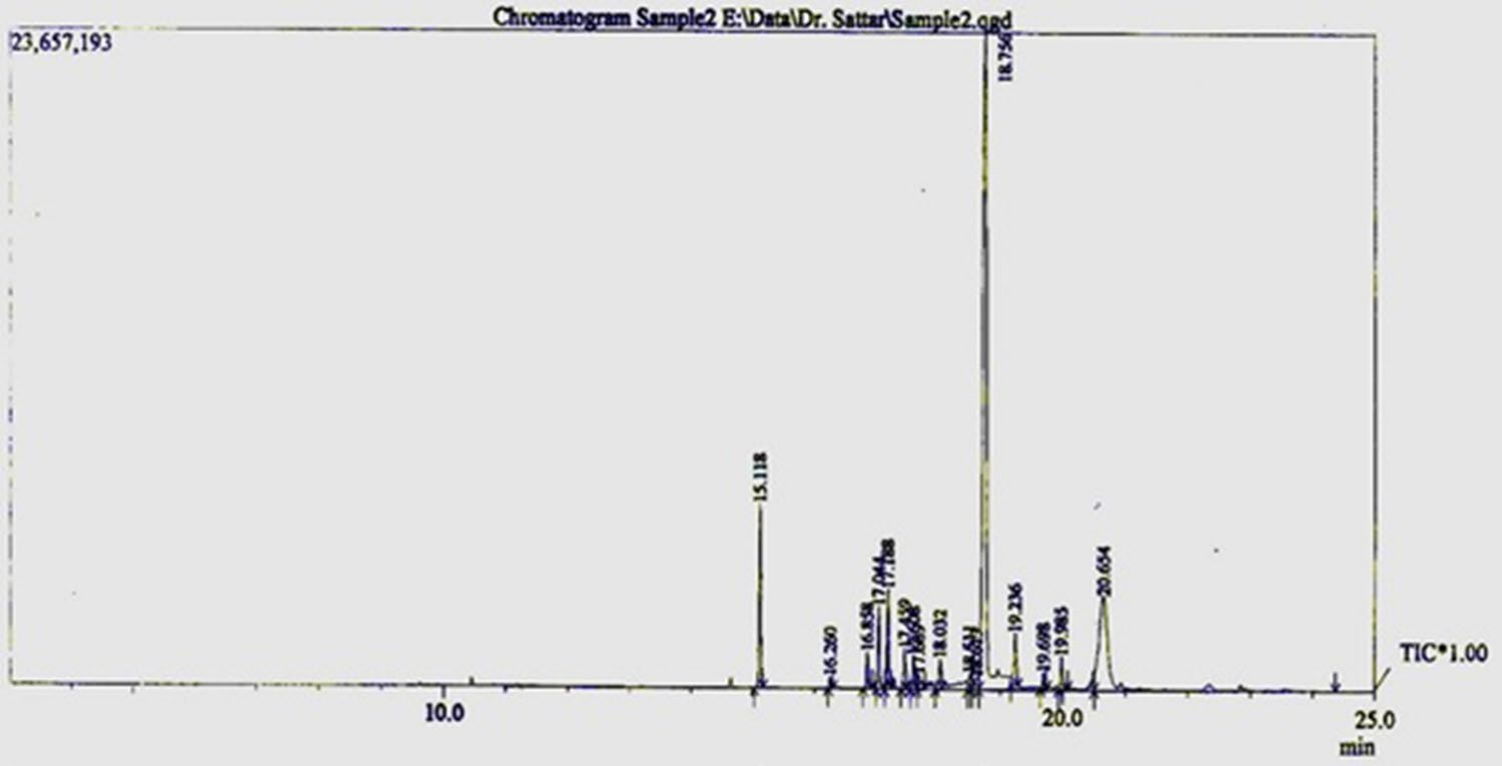
GC–MS chromatogram of MEZZR.

The chemical composition of MEZZR was analyzed by GC–MS. The chromatogram obtained from the MEZZR is presented in Fig. 1. The result of GC–MS analysis revealed that the major components of MEZZR were 1,2-benzenedicarboxylic acid (38.4%), zerumbone (36.9%), α-Cariophyllene (10.6%) (Table 1).

**Table 1:** Chemical constituents of MEZZR.

Peak#	Name of compound	Retention Time	(%) percentage composition
1	Alpha-caryophyllene	15.118	10.640
2	Cyclohexane, 1-methyl-2,4-bis(1-methylethenyl)-	16.261	0.225
3	Caryophyllene oxide	16.858	0.659
4	Beta-selinene	17.044	3.532
5	(-)-Caryophyllene oxide	17.188	2.241
6	(-)-Caryophyllene oxide	17.460	0.822
7	Santolina triene	17.608	1.175
8	Elemol	17.688	0.417
9	Longipinan, Trans-	18.033	0.863
10	Zerumbone	18.510	0.429
11	Cis-Z-.alpha.-bisabolene epoxide	18.614	0.311
12	Zerumbone	18.756	36.997
13	2-propen-1-ol, 3-(2,6,6-trimethyl-2-cyclohexen-1-yl)-	19.237	2.053
14	Veridiflorol	19.699	0.584
15	(-)-Caryophyllene oxide	19.986	0.622
16	1,2-Benzenedicarboxylic Acid	20.652	38.429

### Phytochemical content and antioxidant activity

**Figure 2:**
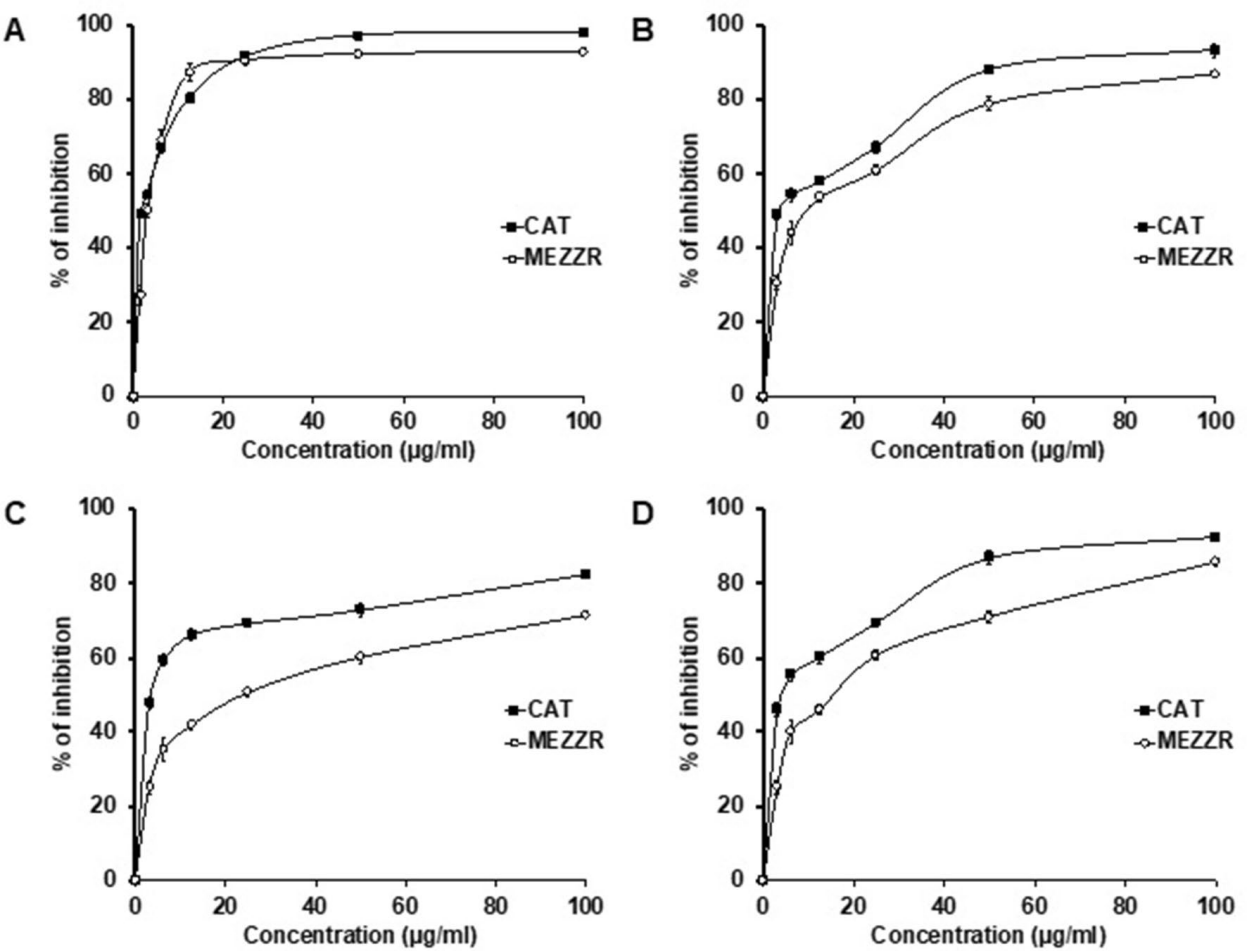
Determination of (**A**) DPPH radical scavenging activity, (**B**) ABTS radical scavenging activity, (**C**) Nitric oxide radical scavenging activity, and (**D**) lipid peroxidation inhibition activity of MEZZR and catechin. Data represents the mean ± SD from three experiments.

As presented in Table 2, the contents of total phenolic and flavonoid of MEZZR were found to be 70.65 ± 0.25 mg/g, 30.40 ± 1.15 mg/g of the dry weight of the extract, respectively. Antioxidant activity of MEZZR was examined by some commonly used in vitro antioxidant assays. Table 2 shows the DPPH, ABTS, nitric oxide, and lipid peroxidation inhibition activities of MEZZR and catechin (CAT). An increased free radical scavenging activity was noticed with the increasing concentrations of MEZZR (Fig. 2A–D). The IC_50_ values of MEZZR and catechin for DPPH, ABTS, nitric oxide, and lipid peroxidation inhibition assays were 3.43 ± 1.25, 11.38 ± 1.39, 23.12 ± 3.39, 16.47 ± 1.47 µg/mL and 2.65 ± 0.98, 4.59 ± 1.13, 3.23 ± 2.68, 7.45 ± 2.00 µg/mL, respectively (Table 2).

**Table 2:** Phytochemical analysis and antioxidant effect of MEZZR.

Sample	TPC (mg of gallic acid equivalent/g of extract)	TFC (mg of catechin equivalent/g of extract)	DPPH radical scavenging activity (IC_50:_µg/ml)	ABTS radical scavenging activity (IC_50:_µg/ml)	Nitric oxide scavenging activity (IC_50_: µg/ml)	Lipid peroxidation inhibition activity (IC_50_:µg/ml)
MEZZR	70.65 ± 0.25	30.40 ± 1.15	3.43 ± 1.25	11.38 ± 1.39	23.12 ± 3.39	16.47 ± 1.47
Catechin	–	–	2.65 ± 0.98	4.59 ± 1.13	3.23 ± 2.68	7.45 ± 2.00

Data are expressed as mean ± SD.

*TPC* Total phenolic content, *TFC* Total flavonoid content, Catechin used as a standard.

### Cytotoxic effect of MEZZR against EAC cells (in vitro)

**Figure 3:**
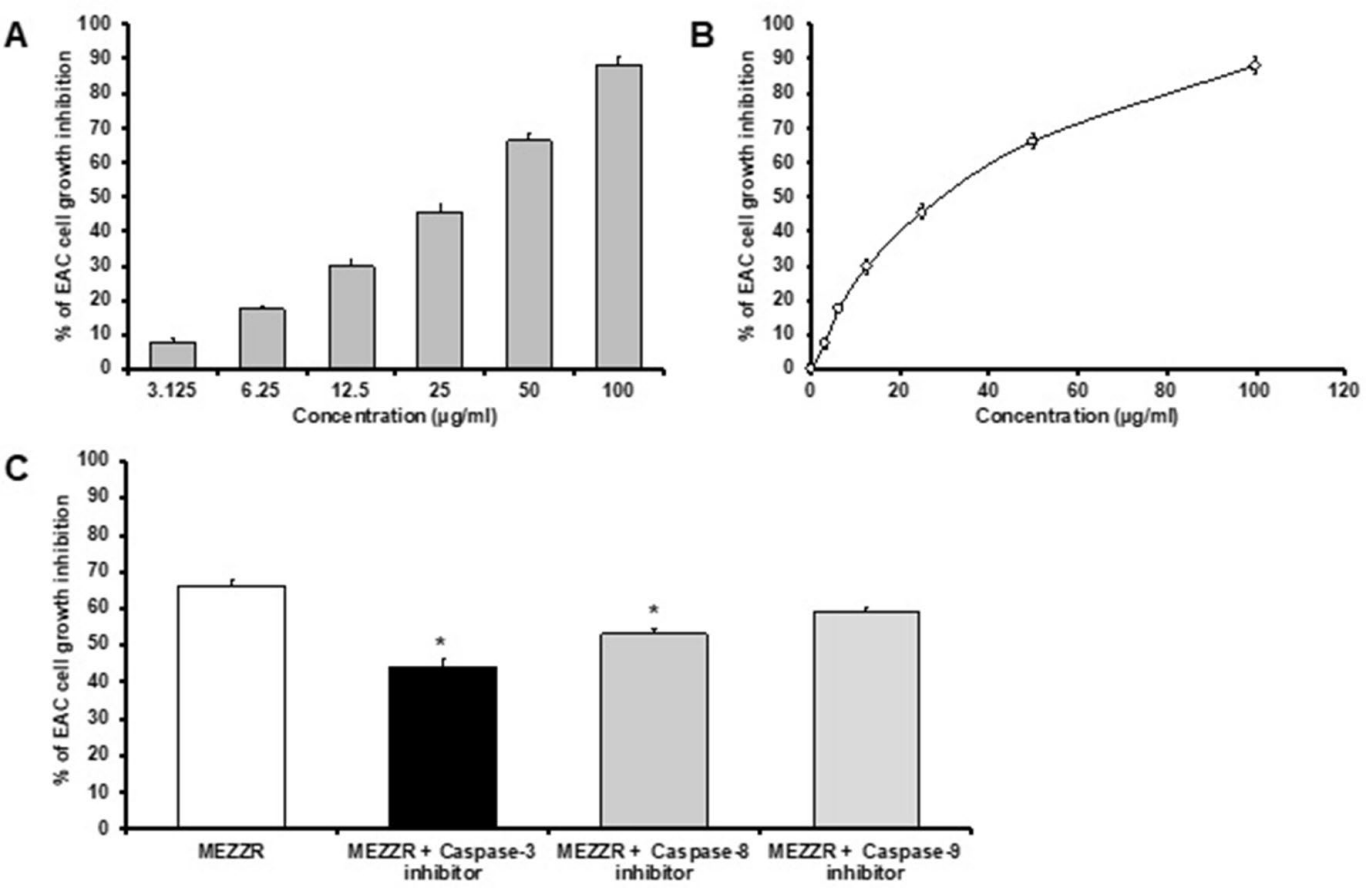
MEZZR inhibits the growth of EAC cells. (A) EAC cells were treated with various doses of MEZZR as indicated for 24 h. The inhibition ratios were measured by the MTT assay (n = 3, mean ± SD). (B) IC_50_ value of MEZZR was calculated from the dose–response curve. (C) EAC cells growth inhibition caused by the MEZZR in the presence of caspase-3, -8 and -9 inhibitors. *p < 0.05, significantly different from MEZZR-treated EAC cells (one-way ANOVA). Data represents the mean ± SD from three experiments.

The inhibitory effect of MEZZR on Ehrlich ascites carcinoma cells growth was evaluated by MTT assay and it explored that the death of EAC cells by MEZZR was happened in a dose dependent manner. Treatment with MEZZR at lowest concentration brought reduction on cancer cell growth that was markedly increased with increasing concentration of MEZZR in respect to control (Fig. 3A). The IC_50_ value of the MEZZR was found to be 27.49 ± 2.25 µg/ml against EAC cell (Fig. 3B).

### MEZZR enhances the antitumor potential on EAC cells

The effect of MEZZR on EAC cell growth on day 7 after EAC transplantation was summarized in Table 3. Maximum cell growth inhibition (58.87%) was found after treatment with 10 mg/kg i.p. per mouse per day, conversely, 69.64% cell growth inhibition was observed at dose 20 mg/kg i.p. per mouse per day. Bleomycin at dose 0.3 mg/kg i.p. showed 92.84% cell growth inhibition. The effect of MEZZR on survival time and weight gain of EAC cell bearing mice was shown in Table 3. The mean survival time of EAC bearing mice was significantly prolonged by administering the MEZZR at both concentrations compared to the EAC control. The increase in the life span of EAC bearing mice treated with MEZZR (10 and 20 mg/kg body weight, i.p.) was found to be 20.23 and 53.40%, respectively, whereas it was 83.72% for the group treated with an anticancer drug, bleomycin (0.3 mg/kg body weight, i.p.). The average weight gain of EAC cell bearing mice was 14.80 ± 0.83 g whereas it was 6.7 ± 0.24, 3.28 ± 0.32 and 2.7 ± 0.65 for the groups treated with MEZZR (10 and 20 mg/kg body weight, i.p.) and bleomycin (0.3 mg/kg body weight, i.p.), respectively.

**Table 3:** Effect of MEZZR on cell growth inhibition, survival time and body weight gain of EAC cell-bearing Swiss albino mice.

Treatment	Viable EAC cells on day 6 after inoculation (× 10^7^ cells/ml)	Percentage (%) cell growth inhibition	Mean survival time; MST (in days)	Percentage increase of life span; % ILS	Body weight gain (g) after 15 days
EAC + Control	5.91 ± 0.19	–	23.8 ± 1.30	–	14.80 ± 0.83
EAC + MEZZR (10 mg/kg)	2.44 ± 0.28*	58.87 ± 4.16	28.6 ± 2.07*	20.23 ± 3.79	6.7 ± 0.24*
EAC + MEZZR (20 mg/kg)	2.22 ± 0.26*	69.64 ± 2.58	36.40 ± 1.14*	53.40 ± 4.07	3.28 ± 0.32*
EAC + Bleomycin (0.3 mg/kg)	0.39 ± 0.09*	92.84 ± 1.68	39.5 ± 0.98*	83.72 ± 3.45	2.7 ± 0.65*

Data are expressed as mean ± SD for six animals in each group. Bleomycin is an anticancer drug. *P < 0.05: against EAC control group.

### MEZZR treatment causes restoration of hematological parameters in EAC cell bearing mice

The effect of MEZZR on the hematological profile of mice bearing the EAC cells are shown in Table 4. On the 12th day, a significant change in the hematological properties of EAC-bearing mice was observed compared to normal mice. The total white blood cell (WBC) count was found to increase with a reduction in the Hb content of red blood cells (RBC) due to tumor induction. At the same time, treatment with MEZZR (10 and 20 mg/kg body weight, i.p.) caused a significant restoration of the total WBC and RBC count and hemoglobin content almost to the normal values.

**Table 4:** Effect of MEZZR on blood parameters of EAC cell-bearing Swiss albino mice.

Treatment	RBC Cells (× 10^9^) /ml^a^	WBC Cells (× 10^6^) /ml^a^	% of Hb gm/dl^a^
Normal mice	6.11 ± 0.40	10.4 ± 1.2	14.48 ± 0.52
EAC + Control	2.36 ± 0.32*	37.00 ± 6.78*	10.86 ± 0.94*
EAC + MEZZR (10 mg/kg)	3.10 ± 0.31*	18.00 ± 2.91*	14.10 ± 0.81*
EAC + MEZZR (20 mg/kg)	4.36 ± 0.41*	8.8 ± 2.58*	15.8 ± 0.54*

^a^Data are expressed as mean ± SD for six animals in each group.

*P < 0.05: against EAC control group.

### MEZZR leads to induction of apoptosis of EAC cells

**Figure 4:**
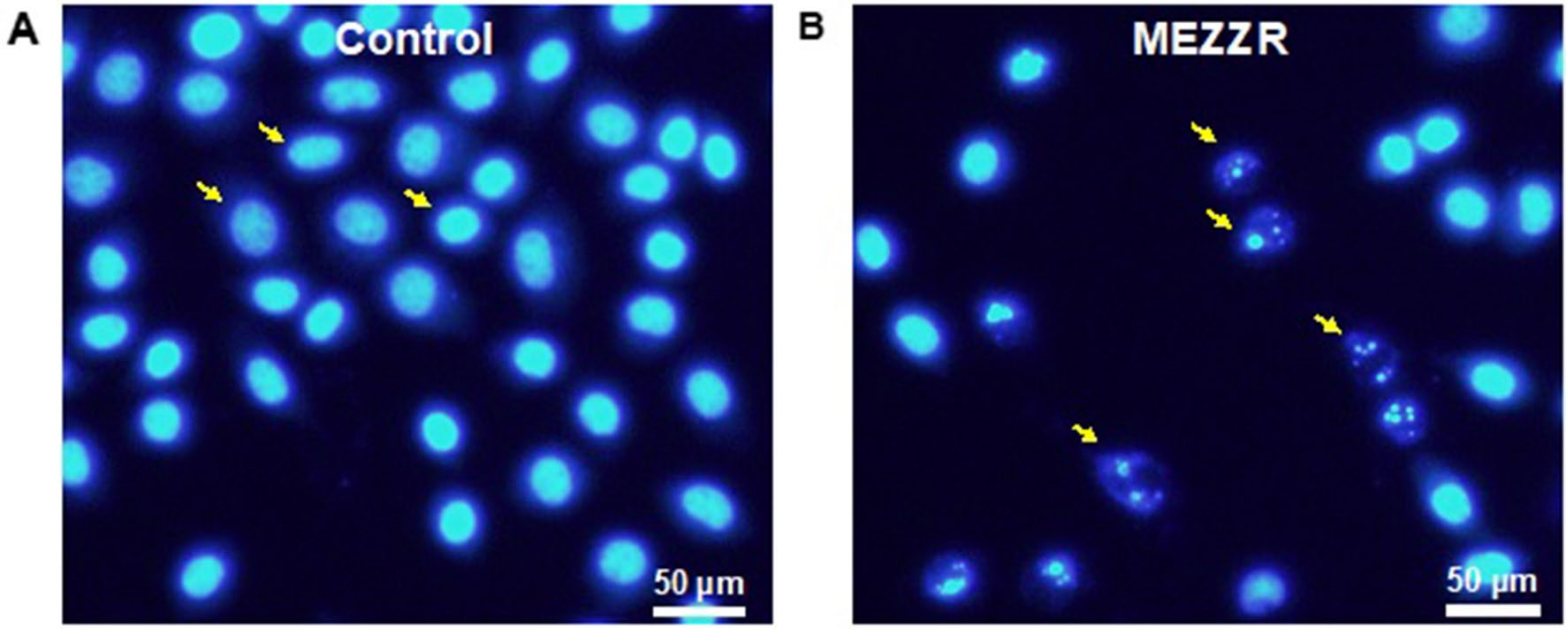
MEZZR triggers apoptosis in EAC cells. EAC cells collected from the treated and non-treated EAC bearing mice were stained with Hoechst 33342 and then cell morphology was observed by a fluorescence microscope. The left panel (**A**) indicates control, and the right panel (**B**) indicates MEZZR (20 mg/kg/day) treatment. Note that apoptotic characteristics, nuclear condensation and fragmentation are shown in (**B**).

We examined whether the antiproliferative effect of MEZZR on EAC cells was due to apoptosis. Apoptosis-inducing effects on EAC cells can be seen in Fig. 4, which shows cell shrinkage, membrane blebbing, chromatin condensation, and nuclear fragmentation in MEZZR-treated EAC cells compared to the untreated controls.

### Effect of caspases on MEZZR-induced cytotoxicity in EAC cells

To investigate the involvement of caspases in the apoptotic cell death of EAC cells induced by the treatment of the MEZZR three caspase inhibitors caspase-3, -8 & -9 were used. Treatment with MEZZR inhibited the growth of EAC cells by 66.19% whereas the inhibition was reduced to 44, 53 and 59% in the presence of caspase-3, caspase-8 & caspase-9 inhibitors, respectively (Fig. 3C).

## Discussion

Free radicals play an imperative role in the pathogenesis of many human ailments including cancer; in contrast, antioxidants play an essential role to protect from these ailments^24^. Most medicinal plants are rich in polyphenols, flavonoids, and flavonols, thus exhibiting considerable antioxidant and anticancer properties^25–30^. In the current study, we found MEZZR to be a rich source of the phenolic and flavonoid compounds that give its antioxidant capacity. The results of this study are in good contract with previous studies on other plant materials^31,32^. Due to the complex nature of phytochemicals, plant extracts free radical scavenging activity cannot be evaluated by a single method alone. In this study, we adopted several free radical generating systems to evaluate the antioxidant capacity of MEZZR. The DPPH and ABTS radical scavenging assays provide a redox-functioned proton ion for unstable free radicals and play a crucial role in stabilizing dangerous free radicals in the animal body^33^. In addition, the normal vascular endothelial cell produces nitric oxide, which plays a critical role in the inflammatory process in the animal body^34^. Free radical-induced lipid peroxidation has recently received much attention owing to its involvement in various pathological conditions including cancer^35^. In this study, MEZZR revealed the potent free radical scavenging activity mediated by DPPH, ABTS, nitric oxide, and inhibition of lipid peroxidation experiments. These results suggest that the presence of phenols and flavonoids in the MEZZR may be responsible for its high antioxidant potency. The results are in good contrast with previous reports on other plant extracts^31,32^ and indicate the probable antitumor efficacy of MEZZR.

Herein, the cytotoxicity activity of the MEZZR was confirmed against the EAC cells by an MTT colorimetric assay. According to the MTT assay, MEZZR caused a concentration-dependent marked growth inhibitory effect in EAC cells following 24 h of exposure. This result is similar to previous studies^31,36,37^. These activities might be due to the presence of phytochemicals such as polyphenols and flavonoids in MEZZR with antioxidant properties. An earlier study revealed a relationship between secondary metabolites and their cytotoxic effect^38^.

The above findings influenced us to evaluate the antitumor activity of the MEZZR against tumor-bearing mice. The result showed that MEZZR inhibited the EAC cells growth, reduced average weight gain and enhanced the survival time of EAC cell-bearing mice, which are used to determine the efficacy of a particular compound as an anticancer agent and provide a reliable basis for evaluating the potency of any anticancer drug^39^. The data are also consistent with those reported previously in the literature^40–44^.

Next, we investigated the anticancer effect of MEZZR by evaluating its impact on changing the hematological parameters of tumor bearing mice. EAC cell-bearing mice have been shown to suffer from anemia due to reduced RBC or Hb production. Iron deficiency or hemolytic/myelopathic conditions may lead to this phenomenon^31^. EAC-bearing mice treated with the MEZZR brought the hematological characteristics closer to normal levels significantly. This similar type of observation was demonstrated in other studies where EAC-bearing mice were treated with bioactive compounds^40–44^. These results indicate that MEZZR has a protective action on the hematological system without inducing myelotoxicity, which is one of the most common acute side effects of cancer chemotherapy.

As a cell-suicidal mechanism, apoptosis is an important process within cell and it is characterized by the change of morphological features such as nuclear fragmentation, membrane blebbing, cell shrinkage and chromatin condensation^45^. Induction of apoptosis is a highly desired characteristic of an anticancer drug since this process helps the drug to remove cancer or malignant cells without damaging normal cells^46,47^. After staining with a blue fluorescing dye like Hoechst 33342, analysis of cells by fluorescence microscope is a rapid and convenient way to observe cell morphological features such as nuclear fragmentation, chromatin condensation^45^. In our study, induction of cell death by MEZZR was confirmed by notable features of apoptosis i.e., chromatin condensation and nuclear fragmentation (Fig. 4) as observed by fluorescence microscopy after staining cells with Hoechst 33342. These findings suggest that MEZZR possesses antiproliferative activity on EAC cells which could be resulted from the induction of apoptosis or inhibition of cell growth. A number of studies conducted previously also reported the induction of apoptosis in EAC cells during the treatment with different plant extracts^31,42^. A series of proteases, known as caspases, are believed to be responsible for the change of morphological features and apoptotic cell death^48^. Treatment of EAC cells with 50 µg/ml for 24 h induced 66.19% cell death which was reduced to 44, 53 and 59% in the presence of caspase-3, caspase-8 & caspase-9 inhibitors, respectively (Fig. 3C) indicating the possible involvement of these caspases in MEZZR-induced EAC cell apoptosis. This result is similar to previous studies^42,49^.

In addition, the presence of zerumbone and 1,2-benzenedicarboxylic acid in MEZZR was confirmed by GC–MS analysis and these compounds were reported to possess antitumor activity^22,50^. Thus, the observed potent antitumor activity of MEZZR in this study is because of the interactive effect of these active compounds and the antioxidative activity of phytochemicals like polyphenols and flavonoids.

Our study has some limitations. We did not identify the active principles that are responsible for the anticancer activity of the rhizome of *Zingiber zerumbet*. In addition, this study did not confirm the molecular mechanism underlying the inhibition of EAC cells by MEZZR. Additional research will be conducted to isolate active constituents from the crude extract and to explore their effects on cancer signaling pathways.

In conclusion, the results derived from the present investigation have shown for the first time that MEZZR dose-dependently caused a significant inhibition of EAC cell proliferation in vitro and in vivo in mice. MEZZR significantly decreased tumor weight in EAC-challenged mice, restored hematological parameters, and enhanced the life span compared to untreated control mice. MEZZR induces apoptosis which was confirmed by observation of change of cell shape and nuclear morphology. It was further confirmed by the caspase-3, -8 and -9 inhibitors. The present study results highlight the promising antioxidant and antitumor effects of MEZZR.

## Materials and methods

### Chemicals and reagents

Hoechst 33342, RPMI-1640 medium, and MTT were purchased from Sigma (USA). Penicillin–streptomycin and fetal calf serum from Invitrogen (USA). Trypan blue and all other chemicals were of analytical grade obtained commercially.

### Plant materials and extraction of *Zingiber zerumbet* rhizome

Fresh rhizomes of *Zingiber zerumbet* (Family: Zingiberaceae) used in this present study were accumulated from the hilly areas of Chittagong, Bangladesh, and identified and confirmed by a taxonomist Dr. Mohammed Yusuf, BCSIR Laboratory, Chittagong, Bangladesh, and a voucher sample (No: 1061) of this collection was retained in the herbarium of this department for future reference. The rhizomes of *Zingiber zerumbet* collected were shade dried for two weeks and reduced to a coarse powder. Approximately 250 g of powder was immersed in 500 mL methanol for 15 days at room temperature followed by filtration as previously mentioned^51^. Afterward, the solvent was evaporated using a rotatory evaporator at 40 °C and kept in a sealed container for subsequent experiments. The crude methanolic extract of rhizomes of *Zingiber zerumbet* (6 g) was designated as MEZZR.

### GC–MS analysis of MEZZR

Separation and identification of the compounds present in the MEZZR were analyzed by gas chromatography/mass spectrometry (GCMS-QP2010S, Shimadzu Kyoto, Japan) equipped with a capillary column (30 m × 0.25 mm × 0.25 μm). The oven temperature was kept at 60 °C for 1 min and raised to 180 °C at a rate of 10 °C /min and held for 1 min. Then, it was increased to 280 °C at a rate of 20 °C /min and kept constant for 15 min. The oven temperature was then decreased to 100 °C before the injection of the next sample. The sample was injected in the split mode and helium was used as a carrier gas. MS scanning was done from 20 to 550 m/z at 2 scans s-1. After obtaining the chromatogram, the mass spectrum of the unknown compounds was identified.

### Estimation of total phenolic and flavonoid content

**Figure 5:**
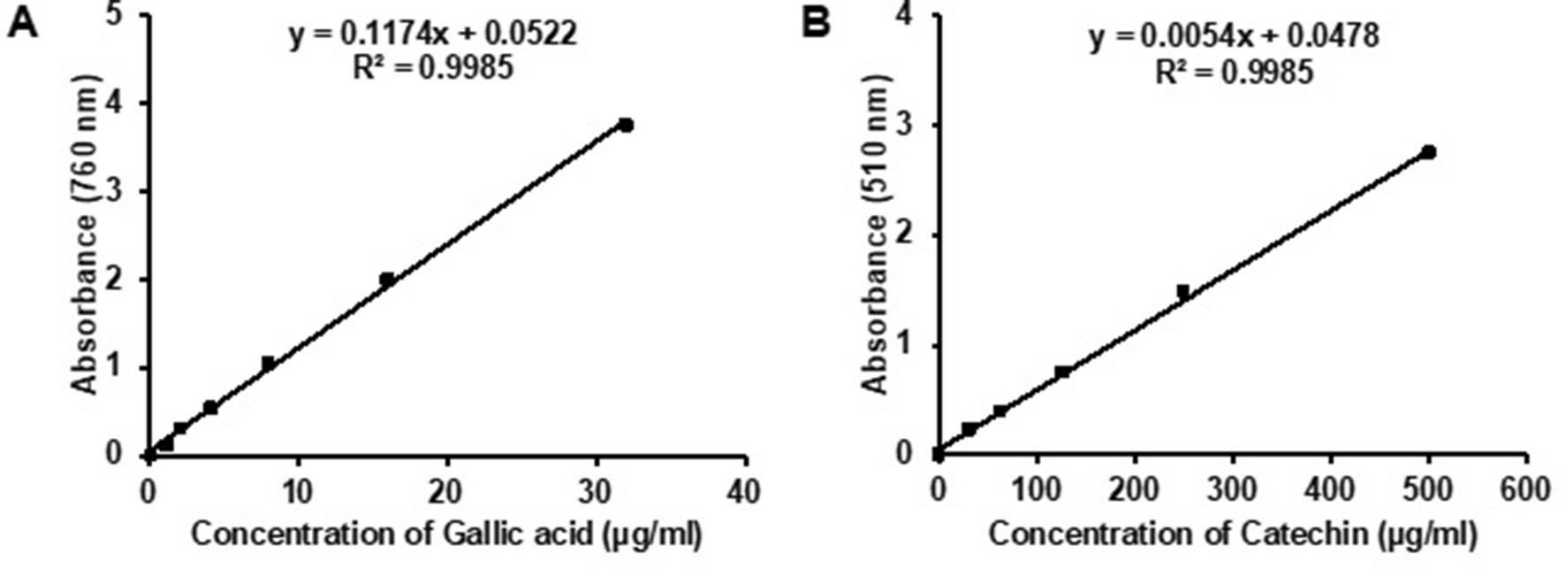
Standard curve of gallic acid (**A**) and catechin (**B**) for determining total phenolics and total flavonoids, respectively. The plot represents the mean ± SD from three experiments.

The total phenolic content in MEZZR was estimated according to the Folin-ciocalteu reagent (FCR) procedure^52^. The calibration curve was prepared by using the gallic acid as standard. The addition of 5 ml of FCR to 1 ml of gallic acid-containing methanol solution or MEZZR was incubated for 5 min at room temperature. After that, 1 ml of 5% (w/v) sodium carbonate (Na_2_CO_3_) solution was added to the reaction mixture. The reaction mixture was made up to 10 ml with distilled water and mixed well. Following incubation for 60 min at room temperature, absorbance was recorded at 760 nm in a spectrophotometer, using methanol as the blank. Total phenolic contents of the extract were calculated based on a standard graph and expressed as mg of gallic acid equivalent/g of dry extract (y = 0.1174x + 0.0522, R^2^ = 0.9985) as shown in Fig. 5A.

The total flavonoid content in MEZZR was quantified as Silva et al. (2015) described by using catechin as a standard for the calibration curve^53^. Briefly, 1 ml of methanol containing 2% (w/v) aluminum chloride solution was combined with the same concentration of test solution. Then, the mixture was vortexed and left standing for 10 min at room temperature. At the end of the incubation, the absorbance of the reaction mixture was recorded at 510 nm in a spectrophotometer, using methanol as the blank. The calibration curve was drawn for catechin (y = 0.0054x + 0.0478, R^2^ = 0.9985) as shown in Fig. 5B. The results were calculated in the same way as described above and expressed as mg of catechin equivalent/g of dry extract.

### DPPH radical scavenging assay

DPPH radical scavenging activity in MEZZR was determined following the previously published protocol^54^. MEZZR at various concentrations (1.5625–100 µg/ml) was mixed with 3 ml of DPPH dissolved in methanol and then the reaction mixture was vortexed thoroughly and kept at room temperature for 30 min away from the light. The absorbance of the reaction mixture was measured at 517 nm in a spectrophotometer, using methanol as the blank. The DPPH radical scavenging capability was calculated by adopting the following equation –

$${\text{\% scavenging effect }} = \frac{{\left( {{\text{Ai}} - {\text{At}}} \right)}}{{{\text{Ai}}}} \times 100$$

where Ai was the absorbance of the control and At was the absorbance of the sample. IC_50_ values (μg/ml), the adequate quantity of the sample required to scavenge 50% DPPH. were determined from the graph which was drawn by plotting percentage scavenging activity against the sample concentrations. Catechin served as the reference standard.

### ABTS radical scavenging assay

The free radical scavenging activity of MEZZR was estimated based on ABTS radical cation decolorization assay^55^. Briefly, 7 mM ABTS in water was mixed with 2.45 mM potassium persulfate (1:1) in the dark at room temperature for 12–16 h to produce ABTS• + solution followed by dilution of the solution to get an absorbance of about 0.70 ± 0.02 at 734 nm wavelength. The ABTS• + solution (3 ml) was added to 1 ml of MEZZR at various concentrations (3.125–100 µg/ml) and mixed well. After standing for 6 min, the absorbance was measured immediately at 734 nm in a spectrophotometer, using methanol as the blank. Subsequently, the percentage of scavenging and IC_50_ values were calculated as narrated in DPPH radical scavenging assay using catechin as standard.

### Nitric oxide radical scavenging assay

The effect of MEZZR on nitric oxide radical was determined according to the previously described method^56^. Briefly, a mixed solution consisting of 2 ml of sodium nitroprusside (5 mM) in phosphate buffer saline (PBS) was added to 1 ml of various concentrations (3.125–100 μg/ml) of MEZZR. The mixture incubated at 25 °C for 150 min was combined with Greiss reagent containing 1% (w/v) sulphanilamide, 2% (v/v) H_3_PO_4_ and 0.1% (w/v) naphthyl ethylenediamine dihydrochloride. The absorbance of the sample was read at 546 nm in a spectrophotometer, using PBS as the blank. The nitric oxide radical scavenging activity of the sample as a percentage and IC_50_ values were judged following the procedure of DPPH assay.

### Determination of Lipid peroxidation inhibition activity assay

Lipid peroxidation inhibition capability of the MEZZR was evaluated according to the method demonstrated by Liu and Ng^57^. In brief, mouse liver was homogenized with a buffer followed by centrifugation to have a liposome. One ml of the reaction mixture prepared by mixing with 0.5 ml of supernatant, 100 μl 10 mM FeSO4, 100 μl 0.1 mM AA and 0.3 ml of different concentrations of the extract from liver was incubated for 20 min at 37 °C followed by addition of 1 ml of 2.8% TCA and 1.5 ml of 1% TBA. The reaction mixture was again heated for 15 min at 100 °C and cooled at room temperature. The absorbance of this mixed solution was taken at 532 nm in a spectrophotometer, using PBS as the blank and the lipid peroxidation inhibition capability of the sample. The standard catechin (expressed as the percentage of inhibition) were enumerated following the equation used in DPPH assay.

### Animals and ethical clearance

Six-week-old female Swiss albino strain mice weighing 26 ± 4 g (International Centre for Diarrheal Disease Research, Bangladesh) were employed for the present study. All mice were allowed free access to water added at *libitium* and standard laboratory animal feed throughout the experimental period. Room temperature and humidity were maintained at 22–28 °C and 55–3%, respectively, with a light–dark cycle of 12 h each. Permission and approval (225/320-IAMEBBC/IBSc) for conducting the experiments on mice were obtained from Experimental Animal Committee of Institute of Biological Sciences, University of Rajshahi, Bangladesh.

### Ehrlich ascites carcinoma cells (EAC)

EAC cells required for this study were kindly provided by the Indian Institute for Chemical Biology (IICB), Kolkata, India, and were maintained in Swiss albino mice by weekly intraperitoneal (i.p.) inoculation of 10^5^ cells per mouse in the research laboratory. In vitro EAC cells were cultured and maintained in RPMI-1640 medium containing 10% fetal calf serum, and 1% penicillin/streptomycin at 37 °C in a humidified cell culture incubator with 5% CO_2_.

### In vitro cell viability test by MTT colorimetric assay

MTT colorimetric assay was conducted to investigate the cell viability of EAC cells as explained in an earlier study^31^. Briefly, EAC cells (2 × 10^4^ cells) were seeded in 200 µl medium/well onto 96-well cell culture plate with different concentrations (3.125–100 µg/ml) of MEZZR and incubated at 37 °C in the presence of 95% air and 5% CO_2_. EAC cells treated with dimethyl sulfoxide (DMSO, 2%) was used as control. Following incubation, aliquot were removed and 180 µl of PBS and 20 µl of MTT (0.1 mg in PBS) were added to each well and again incubated at 37 °C for 8 h. Subsequently, the media of treated cells were removed and 200 µl of isopropanol/HCl was gently added onto each well of the 96-well plate to dissolve the crystals. Following incubation for 1 h at room temperature, the absorbance was taken at a wavelength of 570 nm with a microtiter plate reader. The effects of the MEZZR on the viability of EAC cells was calculated using the following equation -

$${\text{Proliferation inhibition }}\left( {\text{\% }} \right){ } = \frac{{\left( {{\text{A }}{-}{\text{ B}}} \right)}}{{\text{A}}} \times 100$$

where A is the OD_570_ nm of the cellular homogenate without MEZZR and B is the OD_570_ nm of the cellular homogenate with MEZZR.

### Study on MEZZR‐induced cell growth inhibition (in vivo)

EAC cell growth inhibition activity of MEZZR was carried out as explained in a prior study^31^. For this study, Swiss albino normal mice were classified into four groups of six animals (n = 6) each before EAC cells inoculation. On day 0, EAC cells at a density of 1.5 × 10^6^ cells were inoculated intraperitoneally into each mouse of each group. Treatment was commenced after 24 h of EAC transplantation in the mice and continued for 5 consecutive days. Groups 1 and 2 mice were administered intraperitoneally with MEZZR at the doses of 10 and 20 mg/kg/day, respectively. Group 3 was treated with reference anticancer drug bleomycin at the dose of 0.3 mg/kg/day whereas group 4 was used as EAC bearing control receiving 2% DMSO. After 5 days of treatment, EAC cells were harvested with sterile isotonic saline (0.9% w/v, NaCl) from MEZZR treated and control mice and then inverted microscope (XDS-1R, Optika, and Bergamo, Italy) with a haemocytometer was used to count viable cells using trypan blue dye exclusion assay. EAC cells obtained from MEZZR-treated (20 mg/kg/day) and untreated (control) were also used to study the morphological characteristics.

### Studies on morphological appearance of MEZZR treated EAC cells

Morphological changes of EAC cells obtained from MEZZR treated (20 mg/kg/day) and untreated animals were analyzed using nuclear stain, Hoechst 33342. Briefly, the collected EAC cells were washed with PBS and treated with Hoechst 33342 solution (10 µg/ml) at 37 °C for 15 min in the dark to stain nuclei, after which the cells were washed with PBS and observed immediately using fluorescence microscopy (Olympus iX71, Korea) with a 365-nm filter. Cells showing condensed or fragmented nucleus were regarded as apoptotic cells, whereas cells showing intact shape of nucleus were regarded as living.

### Studies on survival time and average weight gain of EAC cell bearing mice

The tumor weight and survival time of MEZZR were carried out by using the method narrated by Ali et al.^31^. In brief, four groups of Swiss albino mice (six mice in each group) were utilized in this study. On day 0, 1.5 × 10^6^ EAC cells per mouse were injected intraperitoneally into the peritoneal cavity of the mouse of each group, and treatment was started following 24 h of post-EAC transplantation and continued for 10 consecutive days. Groups 1 and 2 mice were treated intraperitoneally with MEZZR at the doses of 10 and 20 mg/kg/day, respectively. Group 3 was administered with reference anticancer drug bleomycin at the dose of 0.3 mg/kg/day whereas group 4 was used as EAC bearing control (2% DMSO-treated). To monitor weight gain and survival time, daily alteration of weight and survival time were recorded for each mouse on the 15th day after EAC cell inoculation.

### Studies on hematological parameters

The effect of MEZZR on hematological parameters was examined using a method we described recently^31^. Shortly, mice were divided into four groups (n = 6 per group) and EAC cells (1.5 × 10^6^ cells/mouse) were injected (i.p.) in each mouse of each group at day zero except group 1 (normal control group). Following 24 h post-EAC transplantation, the second and third groups received MEZZR at 10 and 20 mg/kg/day doses, i.p., respectively for 10 consecutive days, whereas group 4 was considered as EAC bearing control (2% DMSO-treated). On day 12, hematological status was estimated from the tail vein blood of each mouse of each group.

### Effect of caspases on MEZZR-induced cytotoxicity in EAC cells

To confirm the involvement of caspases in the MEZZR induced cell death, the untreated EAC cells in RPMI-1640 medium were incubated with Ac-DEVD-CHO (caspase-3 inhibitor, 2 µmol/ml), Ac-IETD-CHO (caspase-8 inhibitor, 2 µmol/ml) and Ac-LEHD-CHO (caspase-9 inhibitor, 2 µmol/ml) for 2 h. Then the cells were treated with 50 µg/ml of MEZZR and incubated for 24 h at 37 °C in CO_2_ incubator. Finally, cell growth inhibition was assessed by MTT assay.

### Statistical analysis

All findings were exhibited as mean ± standard deviation (SD) from no less than three distinct tests. Statistical significance was carried out using one-way ANOVA followed by Dunnett’s t’ test using SPSS software of 16 version and IC_50_ was calculated using GraphPad Prism 6. *P < 0.05 level was considered as statistically significant.

### Experimental research and field studies on plants

All procedures were performed in conformity with applicable institutional, national, and international rules and regulations. Appropriate permissions for collection of *Zingiber zerumbet* was obtained from the competent authority and as per IUCN Policy Statement on Research Involving Species at Risk of Extinction and the Convention on the Trade in Endangered Species of Wild Fauna and Flora.

### Animals and ethical clearance

All the methodological procedures were performed following the relevant guidelines and regulations set by Rajshahi University, Bangladesh, the national and international animal ethical committee. The experiments on mice were carried out as per permission and approval No. 225/320-IAMEBBC/IBSc of the Experimental Animal Committee of Institute of Biological Sciences, University of Rajshahi, Bangladesh and the International Centre for Diarrheal Disease Research, Bangladesh.

### ARRIVE guidelines

The present study is reported in accordance with ARRIVE guidelines.

## Data Availability

All the data is available freely and is included in the manuscript file.

## Abbreviations

CAT: Catechin
MEZZR: Methanol extract of *Zingiber zerumbet* rhizome
GC–MS: Gas chromatography–mass spectrometry
EAC: Ehrlich ascites carcinoma cells
IC_50_: Inhibitory concentration at which 50% inhibition is observed
MTT: 3-(4,5-Dimethythiazol-2-yl)-2,5-diphenyltetrazolium bromide
